# The correlation between serum uric acid and diabetic kidney disease in type 1 diabetes patients in Anhui, China

**DOI:** 10.1186/s12882-023-03302-2

**Published:** 2023-08-24

**Authors:** Jun Jiang, Xiaowan Zhou, Lei Lan, Wei Ren

**Affiliations:** Department of Nephrology, The First Affiliated Hospital of USTC, Division of Life Sciences and Medicine, University of Science and Technology of China, Hefei, 230001 Anhui China

**Keywords:** Type 1 diabetes mellitus, Diabetic kidney disease, Serum uric acid

## Abstract

**Background/Aim:**

To assess the correlation between serum uric acid (UA) level and diabetic kidney disease (DKD) in Type 1 diabetes (T1DM) patients in Anhui, China.

**Methods:**

A total of 231 patients diagnosed with T1DM in our hospital were enrolled between January 2014 and December 2016. Urinary albumin-creatinine ratio (ACR) in patients with hyperuricemia was compared with those without hyperuricemia. The relationship between serum UA level and urinary ACR was examined by Spearman's correlational analysis and multiple stepwise regression analysis. The binary logistic multivariate regression analysis was performed to analyze the correlated factors for type 1 DKD.

**Results:**

The average serum UA levels were 257.7 [215.0, 338.0]μmol/L. The median levels of urinary ACR were significantly higher in patients with hyperuricemia than those without hyperuricemia. In multiple stepwise regression analysis, Serum UA levels were positively correlated with the urinary ACR. The logistic multivariate regression analysis showed that hyperuricemia (OR: 5.24, 95% CI: 1.40—19.65, *P* = 0.014) had an independent positive correlation with DKD in T1DM patients, and the odds of Serum UA to DKD were both elevated as the serum UA levels rose no matter whether adjustment for traditional confounders. The area under the receiver operating characteristic curve was 0.62 (95% *CI*: 0.55–0.70) in assessing the discrimination of the serum UA level for DKD in T1DM patients.

**Conclusions:**

In Chinese patients with T1DM, the serum UA level is positively correlated with urinary ACR and DKD. The correlation between Serum UA and DKD gradually increases with serum UA levels. Serum UA level is not a good predictor for DKD in T1DM patients. Serum UA may directly contribute to initiating DKD, while it has little direct but an indirect effect on an already established DKD in T1DM patients.

**Supplementary Information:**

The online version contains supplementary material available at 10.1186/s12882-023-03302-2.

## Introduction

Diabetic kidney disease (DKD) is one of the most common chronic kidney diseases (CKD), closely related to end-stage renal disease (ESRD) and increased risk of cardiovascular disease (CVD) in type 1 diabetes mellitus (T1DM) [1–3]. The incidence and distribution of T1DM patients in China differed from that in Europe and America [4], and the previous evidence showed that DKD characteristics could be heterogeneous among patients from different ethnicities [5–7]. It is essential to explore the association between modifiable factors and DKD for delaying the progression of DKD in other races.

Hyperuricemia was one of the modifiable factors which were related to cardiovascular disease, CKD, and hypertension [8–10]. The crude and age-standardized prevalence of hyperuricemia was 10.24% and 12.60%, respectively, in the Chinese rural population, [11] while the prevalence in the USA was over 20% [12]. There were already studies on the relationship between serum uric acid (UA) levels and DKD in T1DM patients in European and America. [13–19] Some studies have indicated that elevated serum UA levels were strong and independent predictors of albuminuria and early GFR decline in persons with T1DM, [13–17] the others revealed no causal effects of the Serum UA on the estimated GFR or the risk of CKD in T1DM patients [18, 19]. The association between DKD and serum UA should be further investigated.

Given the heterogeneity of DKD, inconsistent research results of previous studies, and no research focused on the association between serum UA and DKD in T1DM patients in China, it is necessary to analyze further the association between serum UA level and DKD in T1DM patients of China.

## Methods

### Patients

We conducted a cross-sectional study to research the relationship between serum UA level and DKD in T1DM in a medical center in Anhui. A total of 231 hospitalized patients who were considered to have T1DM presenting at the Department of Endocrinology in Anhui Provincial Hospital between January 2014 and December 2016 were recruited. The patients were excluded if they had the following reasons: age under 18 years, no data of the serum UA level, glomerulonephritis, urinary tract infections, active gout, ketosis or ketoacidosis in the 3 months before enrollment. The research ethics committee of Anhui Provincial Hospital approved the study design (NO.2022-RE-331).

### Clinical and laboratory measurements

The methodology of research and data collection has been reported in our published article [20]. We retrospectively collected the demographic information and clinical data using the hospital's database.

### Definitions

Hyperuricaemia was typically reported when serum UA was higher or equal to 420umol/L (7 mg/dl) [9]. The clinical diagnosis of T1DM was based on the American Diabetes Association's descriptions of T1DM [21]. According to Standards of Medical Care in Diabetes -2022 of the American Diabetes Association, DKD was diagnosed [22]. Urine albumin excretions were assessed by urinary ACR in spot urine samples. Normal albuminuria (non-DKD) was defined as a urinary ACR of less than 30 mg/g, while DKD was defined as a urinary ACR greater than or equal to 30 mg/g [22].

### Statistical analysis

All statistical analyses were performed using IBM SPSS Statistics 20.0 version (IBM Corporation, Armonk, NY, USA). A normal distribution of continuous variables was summarized as means ± standard deviation (SD), while a skewed distribution of continuous variables was expressed as medians with interquartile ranges. For continuous data with a normal distribution and a skewed distribution between patients with hyperuricemia and without hyperuricemia, unpaired Student’s *t-*tests and nonparametric tests, respectively, were used for statistical analyses. The number (n) and percentage (%) in each category were calculated for categorical variables. The categorical variables were evaluated with a *chi-square* test.

The relationship between serum UA level and urinary ACR was performed by Spearman’s correlational analysis and multiple stepwise regression analysis. The binary logistic multivariate regression analysis (Forward: LR) was performed to analyze the correlated factors for DKD in T1DM and calculate the odds ratio (OR) and 95% confidence interval (CI). A *P*-value < 0.05 was considered statistically significant. The predicting value of serum UA level for DKD was evaluated by the area under the receiver operating characteristic curve (AUROC) for discrimination and Hosmer–Lemeshow goodness-of-fit test for calibration. The discrimination was used to evaluate the ability of a factor to identify disease occurrence or not. AUROC > 0.70 implied that the factor had higher discrimination. The calibration was used to evaluate the disease prediction accuracy of a factor. The calibration was higher when P > 0.05 in Hosmer–Lemeshow goodness-of-fit test.

## Results

### Patient characteristics and comparison of baseline covariates

The baseline characteristics of the 231 patients are shown in Table 1. The average serum TC levels, LDL-c levels, and proportion of DKD in patients with hyperuricemia were significantly higher than those without hyperuricemia (*P* < 0.05). The median duration of DM, median serum TG, serum Cr, and urinary ACR levels were higher in patients with hyperuricemia (*P* < 0.05). In contrast, the median eGFR was higher in patients without the hyperuricemia group (*P* < 0.05).

**Table 1:** Patient demographics and laboratory data

Characteristics	Total Participants (*n* = 231)	Patients with Hyperuricaemia (*n* = 29)	Patients without Hyperuricaemia (*n* = 202)	*P*-value
Male/Female	117/114	18/11	99/103	0.19
Age (years)	34.1 ± 11.6	34.7 ± 11.9	34.0 ± 11.6	0.76
Duration of diabetes (years)	6.0 [1.5, 10.0]	10.0 [4.0, 12.0]	5.5 [1.0, 10.0]	**0.013**
BMI (Kg/m^2^)	20.3 ± 3.3	20.2 ± 3.0	20.3 ± 3.3	0.97
HbA1c (%)	9.5 ± 2.7	10.1 ± 3.6	9.4 ± 2.6	0.28
SBP (mmHg)	123.3 ± 17.8	126.9 ± 17.7	122.8 ± 17.8	0.25
DBP (mmHg)	78.8 ± 11.1	80.3 ± 14.5	78.6 ± 10.6	0.43
Hb (g/L)	125.5 ± 19.0	118.9 ± 26.3	126.4 ± 17.6	0.15
TC (mmol/L)	4.3 ± 1.1	4.9 ± 1.3	4.3 ± 1.0	**0.008**
TG (mmol/L)	0.95 [0.64, 1.33]	1.45 [0.93, 2.92]	0.91 [0.63, 1.24]	**0.001**
HDL-C (mmol/L)	1.2 ± 0.4	1.2 ± 0.4	1.2 ± 0.4	0.50
LDL-C (mmol/L)	2.4 ± 0.9	2.8 ± 1.1	2.3 ± 0.8	**0.01**
ALB (g/L)	38.9 ± 5.3	38.9 ± 7.3	38.9 ± 5.0	0.97
UA (μmol/L)	257.7 [215.0, 338.0]	488.0 [460.8, 551.0]	245.5 [205.25, 292.75]	** < 0.001**
Cr (μmol/L)	67 [53, 86]	115 [86, 165]	63 [51, 78]	** < 0.001**
eGFR(ml/min/1.73m^2^)	113.6 [86.3, 147.5]	65.3 [35.2, 101.1]	116.2 [91.5, 149.2]	** < 0.001**
urinary ACR (mg/g)	22.96 [11.1, 73.67]	44.9 [24.9, 1533.4]	21.6 [10.3, 49.9]	** < 0.001**
DKD [n(%)]	93 (40.3%)	22 (75.9%)	71 (35.1%)	** < 0.001**

*ACR* albumin–creatinine ratio, *ALB* albumin, *BMI* body mass index, *Cr* creatinine, *DBP* diastolic blood pressure, *eGFR* estimated glomerular filtration rate, *Hb* hemoglobin, *HbA1c* glycated hemoglobin A1c, *HDL-C* high-density lipoprotein cholesterol, *LDL-C* low-density lipoprotein cholesterol, *SBP* systolic blood pressure, *TC* total cholesterol, *TG* triglyceride, *UA* uric acid, *DKD* diabetic kidney disease

### Correlations between serum UA levels and other characteristics

To analyze the factors associated with serum UA, the correlations between serum UA levels and other characteristics were assessed by Spearman's correlation analysis. Table 2 shows that serum UA level is positively correlated with the duration of DM, serum TG concentrations, and urinary ACR and negatively associated with eGFR (all* P* < 0.05). After adjustment for those significant factors, stepwise multiple linear regression analysis showed an independent positive association between serum UA levels and urinary ACR and a negative correlation between serum UA levels and eGFR(both* P* < 0.05).

**Table 2:** The related factors of serum UA level in patients with T1DM

Parameter	Spearman’s correlation analysis		Stepwise multiple linear regression	
	r	*P*-value	β Coefficient ± SE	*P*-value
Age (years)	- 0.007	0.91		
Duration of diabetes (years)	0.25	< 0.001		
BMI (kg/m2)	-0.076	0.27		
HbA1c (%)	-0.104	0.12		
SBP (mmHg)	0.083	0.21		
DBP (mmHg)	0.048	0.47		
Hb (g/L)	0.046	0.49		
TC (mmol/L)	0.11	0.11		
TG (mmol/L)	0.263	< 0.001	20.9 ± 5.8	< 0.001
HDL-C (mmol/L)	-0.09	0.20		
LDL-C (mmol/L)	0.11	0.11		
ALB (g/L)	0.049	0.46		
Urinary ACR (mg/g)	0.189	0.004	0.02 ± 0.01	0.008
eGFR (ml/min/1.73m^2^)	-0.459	< 0.001	-0.8 ± 0.1	< 0.001

*ACR* urinary albumin–creatinine ratio, *SE* standard error, *ALB* albumin, *BMI* body mass index, *DBP* diastolic blood pressure, *eGFR* estimated glomerular filtration rate, *Hb* hemoglobin, *HbA1c* glycated hemoglobin A1c, *HDL-C* high-density lipoprotein cholesterol, *LDL-C* low-density lipoprotein cholesterol, *SBP* systolic blood pressure, *TC* total cholesterol, *TG* triglyceride, *UA* uric acid

### The independent and positive association between serum UA level and DKD

The correlations between urinary ACR and other characteristics have been reported in our published article [20]. Urinary ACR was correlated with eGFR, Hb levels, ALB, the duration of diabetes, HbA1c, SBP, DBP, TC, TG, LDLC, UA, and Cr concentrations in Spearman's correlation analysis. The binary logistic regression multivariate analysis included these urinary ACR-correlated factors to identify the association between serum UA level and DKD. When DKD was set as the dependent variable, the duration of diabetes, Hb level, eGFR, ALB, HbA1c, SBP, DBP, TC, TG, LDL-C, UA, and Cr concentrations were set as covariates. Table 3 shows that in multivariate analysis (Forward: LR), Hyperuricaemia (OR: 5.24, 95% CI: 1.40—19.65, *P* = 0.014) is independent positively correlated with DKD in T1DM patients, while the other independent related factors for DKD in patients with T1DM are the Hb level (OR: 0.94, 95% CI: 0.91- 0.96, *P* < 0.001), duration of DM (OR: 1.14, 95% CI: 1.07—1.22, *P* < 0.001), HbA1c (OR: 1.42, 95% CI: 1.19—1.68, *P* < 0.001), DBP (OR: 1.07, 95% CI: 1.04—1.11, *P* < 0.001) and TG (OR: 1.67, 95% CI: 1.06—2.62, *P* = 0.027).

**Table 3:** Logistic regression multivariate analysis of independent related factors for the diabetic kidney disease in 231 patients with type 1 diabetes

Variable	OR (95% CI)	*P*
Hyperuricaemia (yes *vs*. no)	5.24 (1.40—19.65)	0.014
Duration of diabetes (years)	1.14 (1.07 -1.22)	< 0.001
HbA1c (%)	1.42 (1.19 -1.68)	< 0.001
DBP (mmHg)	1.07 (1.04 -1.11)	< 0.001
Hb (g/L)	0.94 (0.91- 0.96)	< 0.001
TG (mmol/L)	1.67 (1.06 -2.62)	0.027

*OR* odds ratio, *CI* confidence interval, *UA* uric acid; HbA1c, glycated hemoglobin A1c, *DBP* diastolic blood pressure, *Hb* hemoglobin, *TG* triglyceride

Serum UA levels were stratified to explore further the association between serum UA levels and DKD in T1DM patients (Table 4). Table 4 shows that serum UA level positively correlates with DKD in T1DM patients in both continuous and categorical variables, even after adjusting for age, sex, duration of diabetes, and HbA1c. The odds of Serum UA to DKD were both elevated as the serum UA level rose no matter whether adjustment for traditional confounders. The univariate regression model for each sex was also performed. The results showed that serum UA level both positively correlates with DKD in T1DM patients in each gender, while correlation coefficients in female T1DM patients were higher than that in male patients (Supple Table 4– 1 and 4– 2). When UA ≥ 420 μmol/L in female T1DM patients, all patients were DKD, and none were non-DKD. The multivariate logistic regression analysis was not performed due to the limited samples of DKD in male patients and non-DKD patients in female patients.

**Table 4:** The relationship between serum uric acid levels and diabetic kidney disease in type 1 diabetes patients

**Model**		**UA (μmol/L)**	**Unadjusted**		**Adjusted**	
			**OR (95% CI)**	***P*-value**	**OR (95% CI)**	***P*-value**
Model 1	UA (continuous)	**—**	1.004(1.002–1.007)	< 0.001	1.005(1.002–1.007)	0.001
Model 2	UA (categorical)	< 360 μmol/L	1 (Reference)			
		≥ 360 μmol/L	3.02 (1.56–5.86)	0.001	2.75 (1.26—6.03)	0.011
Model 3	UA (categorical)	< 420 μmol/L	1 (Reference)			
		≥ 420 μmol/L	5.80 (2.36–14.24)	< 0.001	5.44 (1.95 -15.19)	0.001

*UA* uric acid, *CI* confidence interval, *OR* odds ratio, Model 1, Model 2, and Model 3 adjusted for age, sex, the duration of diabetes, glycated hemoglobin A1c

**Fig. 1:**
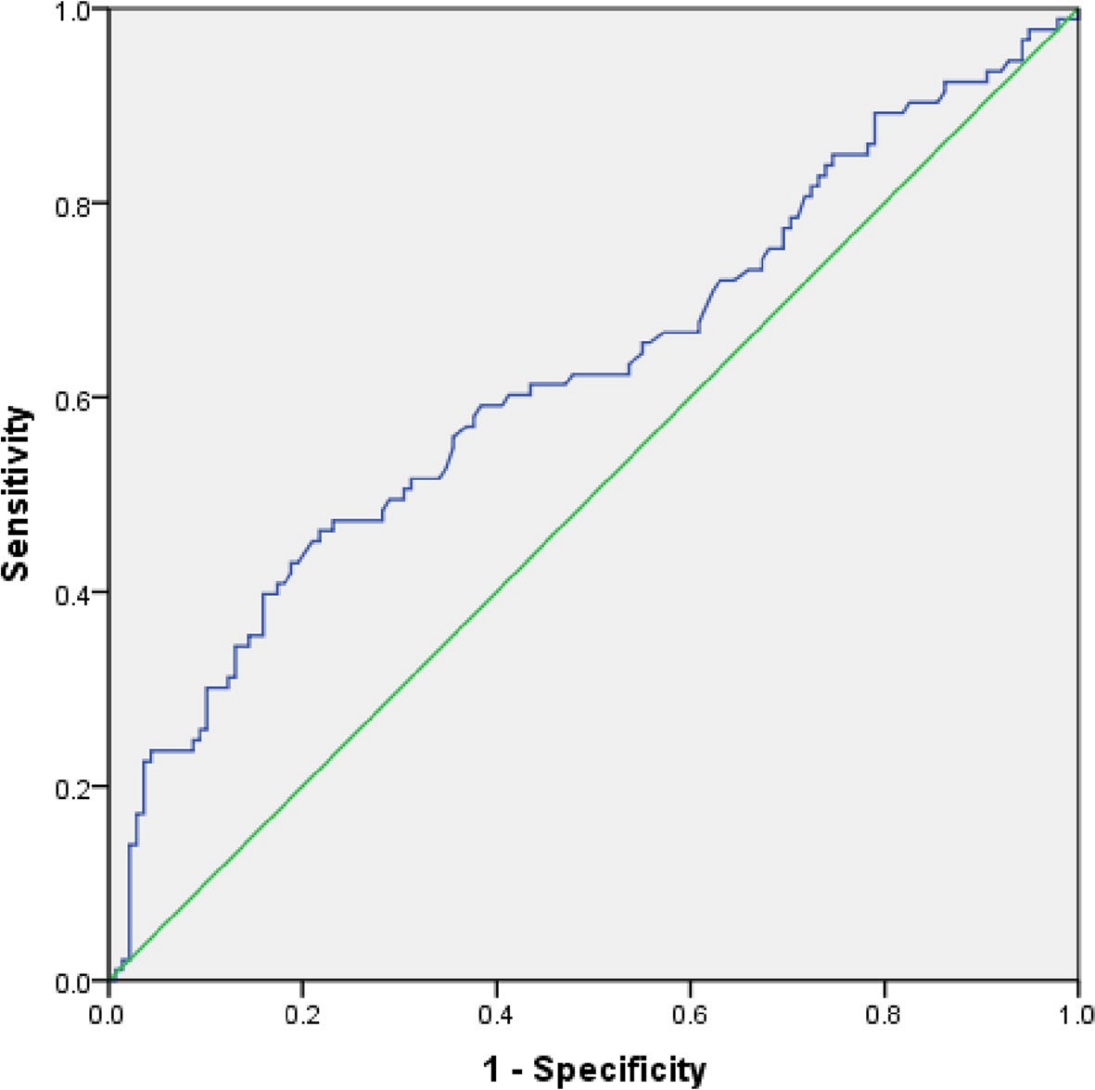
Receiver operation characteristic curve analysis to assess the discrimination of the serum uric acid level for type 1 diabetic kidney disease

To further investigate the predicting value of UA level for DKD in T1DM patients, Hosmer–Lemeshow goodness-of-fit test was used to value the calibration, and the AUROC was used to assess the discrimination. Figure 1 shows that the AUROC is 0.62 (95% CI: 0.55–0.70) in assessing the discrimination of the serum UA level for DKD. The value of *P* was 0.65 in Hosmer–Lemeshow goodness-of-fit test. The AUROC was only 0.62 and less than 0.70, which implied that the serum UA level was not a good predictor for type 1 DKD.

## Discussion

DKD is one of the most common chronic kidney diseases (CKD) and remains the leading cause of morbidity and mortality in T1DM patients. Meanwhile, microalbuminuria can predict the progression of DKD in T1DM patients [23]. Still, the evidence must be focused on the association between serum UA and DKD in T1DM patients in China. In this present cross-sectional research, we explored if serum UA level was related to the prevalence of type 1 DKD in China. We discovered that the Serum UA level was independently and positively associated with TG and albuminuria and negatively correlated with eGFR. Hyperuricemia was an independent related factor for DKD in patients with T1DM in China, consistent with former research. We also found that the odds of Serum UA to DKD were both elevated as the serum UA level rose no matter whether adjustment for traditional confounders (Table 4). These results further confirmed the association between serum UA levels and DKD. We also found that the serum UA level positively correlated with DKD in T1DM patients in each gender, while correlation coefficients in female T1DM patients were higher than in male patients (Supple Table 4– 1 and 4–2).

Previous studies revealed that high serum UA might contribute to initiating arterial hypertension while it has little effect on already-established hypertension [24]. Several animal research revealed that hyperuricemia promotes the development of hypertension to accelerate renal function deterioration [25, 26]. We speculated that early phases of hyperuricemia contributed to hypertension. This association gradually weakened to disappear even though the hyperuricemia persisted. This may partially be explained that the serum UA level had no correlation with blood pressure, neither SBP nor DBP in our study.

Although hyperuricemia was not correlated with hypertension, it was positively correlated with high TG levels in this research (Table 2). This article also found that the average serum TC and LDL-c levels in patients with hyperuricemia were significantly higher than in patients without hyperuricemia group. In contrast, the average BMI, serum ALB and HbA1c had no significant difference between the two groups (Table 1). This suggested that the correlation between hyperuricemia and hyperlipemia may be independent of patients' blood glucose levels and nutritional status. As we all know, hyperlipemia is an independent risk factor of DKD in T1DM patients and cardiovascular disease (CVD). [27, 28] At the same time, serum uric acid to HDL-cholesterol ratio was associated with hypertension and eGFR [29, 30]. This is easy to understand that hyperuricemia and hyperlipemia are related to CVD and DKD in previous studies [8–10]. Our study also confirmed that TG and hyperuricemia were positively correlated with DKD (Table 3). The effect and mechanism of TG and hyperuricemia on DKD in T1DM patients are worthy of further investigation.

However, the Preventing Early Renal Loss in Diabetes (PERL) trial showed no significant benefits of serum UA reduction with allopurinol on kidney outcomes among patients with T1DM and early-to-moderate DKD, in which the mean serum uric acid was 6.1 mg/dl, and the included patients had a long course of disease: the mean age 51.1 years, the mean duration of diabetes 34.6 years, and the renal complications (most eGFR < 90 ml/min/1.73m^2^) [31]. These findings can not explain the association between serum UA levels and DKD. We speculated that serum UA level might directly contribute to initiating DKD, while it has little direct effect but an indirect effect on an already established DKD in T1DM patients. This could explain the positive association between serum UA levels and DKD and also explain that serum UA levels could not predict type 1 DKD (Fig. 1 showed that AUROC was only 0.62 in assessing the discrimination of the serum UA level for DKD) and no significant benefits of serum UA reduction with allopurinol on kidney outcomes in the PERL study. For timely interventions in the early phases of hyperuricemia may prove more beneficial than treatment at later stages, the PERL study did not enroll enough hyperuricemia patients and may miss the best opportunity for intervention. [8, 24] In addition, the FEATHER trial found that stage 3 CKD patients with hyperuricemia, who had no proteinuria and a higher baseline renal function, got significant benefits in renal outcomes from lowering Serum UA [32]. These results of the FEATHER trial also confirmed our guess. It is suggested that trials enrolling patients at an earlier stage of T1DM with hyperuricemia could lead to different conclusions from the PERL studies.

Some research has revealed the possible mechanism of serum UA levels on DKD. First, serum UA may directly induce immune system activation and alters the characteristics of resident kidney cells to promote renal inflammation, promote interstitial fibrosis and chronic kidney disease development [33–36]. The novel inflammatory predictor, such as C-reactive protein to serum albumin ratio, is higher in diabetic nephropathy [37]. Additionally, several animal research revealed that hyperuricemia promoted the development of hypertension to accelerate renal function deterioration [25, 26]. Hyperuricemia could accelerate renal function deterioration via high systemic blood pressure and cyclooxygenase-mediated, thromboxane-induced vascular disease [38]. Experimental studies demonstrated high serum uric acid levels promoted medial thickening of preglomerular arterioles and were directly correlated with glomerular capillary pressure [39], which led to ischemia and hypoxia, and tubulointerstitial fibrosis [40]. Y Lytvyn et al*.* suggested that plasma UA-mediated afferent arteriolar resistances of patients with T1DM may be caused by the thickening of the afferent renal arteriole, potentiating renal injury by causing renal microcirculation ischemia [41]. Y Lytvyn et al*.* also found that UA lowering in patients with T1DM lowered systolic BP and modulated the renal efferent resistance responses to hyperglycemia but without impacting the RAAS or NO levels, suggesting that plasma UA may augment other hemodynamic or inflammatory mechanisms that control the renal response to hyperglycemia at the efferent arteriole. [42] Furthermore, serum UA levels in the normal range could decrease endothelium-dependent reactions associated with T1DM, and serum UA levels were associated with microvascular endothelial dysfunction in patients with Type 1 DM [43].

However, this research had several limitations. First, our study was just a single-center, cross-sectional observational research, and the power of this result was limited. Second, the conclusions could not exclude the influence of the confounding factors (e.g., RAAS inhibitors, drugs lowering serum UA), and our study did not analyze the relationship between the trend of serum UA level with DKD and the mechanism that serum UA on DKD. Third, whether our research findings suit other groups remain to be determined. The effect and mechanism of TG and hyperuricemia on DKD in T1DM patients are worthy of further investigation. And adequately powered, randomized controlled trials (RCT) with the early stage of T1DM with hyperuricemia are still needed to investigate whether these patients could benefit from lowering serum UA on renal outcomes.

## Conclusion

In Chinese patients with T1DM, the serum UA level is positively correlated with urinary ACR and DKD. The correlation between Serum UA levels and DKD gradually increases with serum UA levels. Serum UA level is not a good predictor for DKD in T1DM patients. We presume that serum UA may directly contribute to initiating DKD, while it has little direct effect but an indirect effect on an already established DKD in T1DM patients.

### Supplementary Information

**Additional file 1:** **Supple Table 4-1. **The relationship between serum uric acid levels and diabetic kidney disease in Female type 1 diabetes patients.** Supple Table 4-2. **The relationship between serum uric acid levels and diabetic kidney disease in Male type 1 diabetes patients.

## Data Availability

The datasets used and/or analysed during the current study available from the corresponding author on reasonable request.
