# Supplementary material for: The correlation between serum uric acid and diabetic kidney disease in type 1 diabetes patients in Anhui, China
Source: BMC Nephrol. 2023 Aug 24;24:252. doi: 10.1186/s12882-023-03302-2 (PMC10463645; doi:10.1186/s12882-023-03302-2)
Supplement: Supplementary file 1 — Additional file 1: Supple Table 4-1. The relationship between serum uric acid levels and diabetic kidney disease in Female type 1 diabetes patients. Supple Table 4-2. The relationship between serum uric acid levels and diabetic kidney disease in Male type 1 diabetes patients. [file 12882_2023_3302_MOESM1_ESM.docx]

**Supple Table 4-1. The relationship between serum uric acid levels and diabetic kidney disease in Female type 1 diabetes patients**

| **Model** |  | **UA (μmol/L)** | **Unadjusted** | |
| --- | --- | --- | --- | --- |
|  |  |  | **OR (95% CI)** | ***P*-value** |
| Model 1 | UA(continuous) | **—** | 1.008(1.004-1.013) | < 0.001 |
| Model 2 | UA(categorical) | <360 μmol/L | 1 (Reference) |  |
|  |  | ≥360 μmol/L | 9.821 (2.129-45.316) | 0.003 |
| Model 3 | UA(categorical) | <420 μmol/L | 1 (Reference) | — |
|  |  | ≥420 μmol/L | — | — |

Note: UA, uric acid; CI, confidence interval; OR, odds ratio. When UA ≥420 μmol/L in female T1DM patients, all patietns were DKD, and none were non-DKD.

**Supple Table 4-2. The relationship between serum uric acid levels and diabetic kidney disease in Male type 1 diabetes patients**

| **Model** |  | **UA (μmol/L)** | **Unadjusted** | |
| --- | --- | --- | --- | --- |
|  |  |  | **OR (95% CI)** | ***P*-value** |
| Model 1 | UA(continuous) | **—** | 1.004 (1.001-1.008) | 0.025 |
| Model 2 | UA(categorical) | <360 μmol/L | 1 (Reference) |  |
|  |  | ≥360 μmol/L | 2.800 (1.169-6.708) | 0.021 |
| Model 3 | UA(categorical) | <420 μmol/L | 1 (Reference) | — |
|  |  | ≥420 μmol/L | 4.651 (1.627-13.299) | 0.004 |

Note: UA, uric acid; CI, confidence interval; OR, odds ratio.
